# Robotics fulfil a strategic need

**DOI:** 10.1155/S1463924691000020

**Published:** 1991

**Authors:** G. L. Taylor, T. R. Smith, G. J. Kamla

**Affiliations:** Shell Development Company Houston Texas USA


Journal of Automatic Chemistry, Vol. 13, No. 1 (January–February 1991), pp. 3–7

### Robotics fulfil a strategic need*

**G. L. Taylor, T. R. Smith and G. J. Kamla**
*Shell Development Company, Houston, Texas, USA*

The authors' jobs in Shell involve responsibility for the analytical activity which includes about 220 people at the central research laboratory and roughly another 50 or so in quality control laboratories at various plants and refineries.

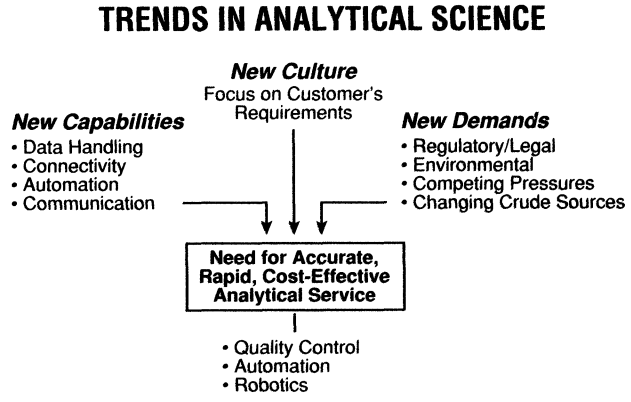

*Figure 1.*

The analytical activity is rapidly changing (figure 1). Analytical chemistry is being revolutionized by new capabilities conferred by the computer. At the same time, business has to face a new set of environmental and regulatory demands, plus new competing pressures. Concurrent with this is a culture stressing quality and continual improvement, and in-depth fulfilment of customers' requirements. In analytical chemistry, this translates to a need for more accurate, more rapid and more cost-effective analytical service.

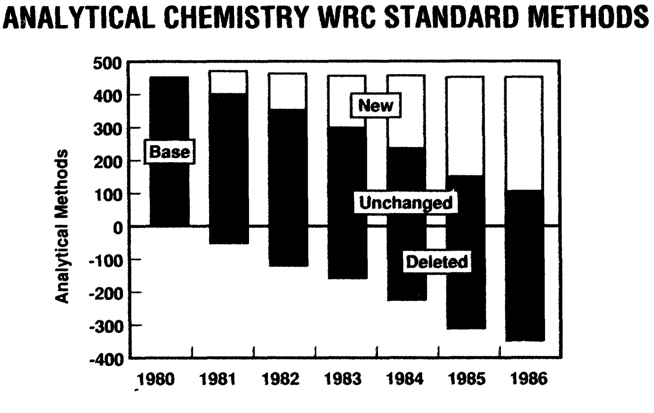

*Figure 2.*

Because it is a rapidly evolving technology, the chemists' arsenal of analytical methods continues to change (figure 2). Figure 2 represents the authors' experience at the research facility during the 1980s. It is clear that without continual modernization of methods and procedures, a large number of methods would be obsolete within a relatively short time. The rate of change is less marked for the quality assurance /quality control type activities, but even these are not exempt from change.

At the same time, a growing concern about exposure to chemicals has spawned a large number of regulations on health and safety (figure 3). Over the last few decades, maximum limits of exposure to materials like benzene have decreased by a factor of 100, and acronyms like OSHA, HAZCOM, and TOSCA have all become familiar words.

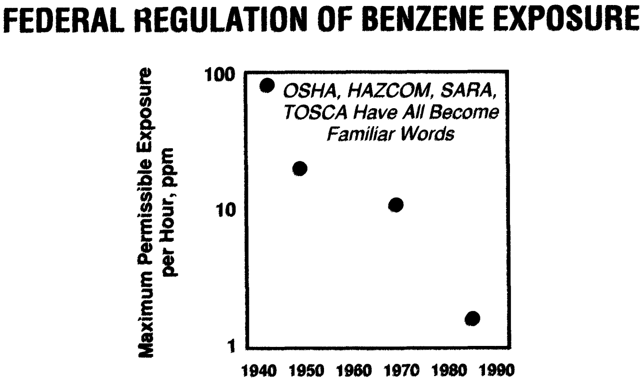

*Figure 3.*

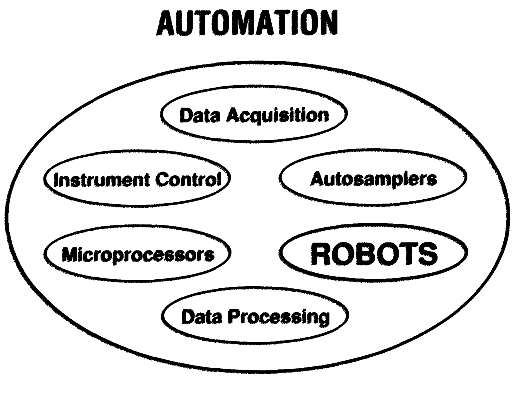

*Figure 4.*

* Abstract published in *Journal of Automatic Chemistry*, Vol. 12, No. 6.

For all of these reasons, automation has become a very high strategic priority in managing analytical futures. It is exactly in line with the need for increased timeliness, precision, and cost effectiveness of analyses, and in addition it tends to minimize exposure to hazards, and allow one to retain flexibility in deployment of human resources.

**SAFETY, PRODUCTIVITY, AND PRECISION**
**IMPROVEMENT THROUGH LABORATORY AUTOMATION**

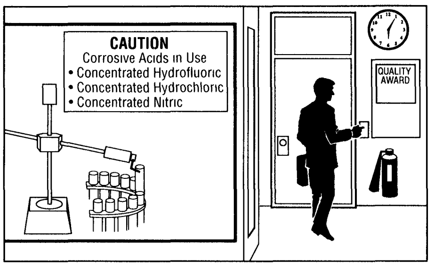

- The First Robot at Shell Has Handled Over 40,000 Samples Since 1984
- Payback Periods Have Been 6 Months or Less for Several Applications
- Some Environmental Applications Have Been Successfully Duplicated at Operating Locations
- Robotic Applications Have Helped to Minimize Worker Exposure to Hazardous Chemicals and Reagents

*Figure 5.*

There are many forms of laboratory automation which will do this successfully and these are shown in figure 4. Robots are probably the most complex of these approaches, and should be used only when the other forms of automation will not suffice. However, when a robot is used (figure 5), one can reasonably expect to see dramatic improvements in safety, productivity and precision. Even more important is the improvement in quality of life for the employees, since robots do best those kinds of jobs which are seldom interesting or challenging for humans.

*Table 1. UV active component analysis.*

| | Manual | Robotic |
|---|---|---|
| Time | 3 hours | 30 minutes |
| Cost | $250 | $70 |
| Precision | 1% | 0.3% |
| Turnaround | 2 months | 2 weeks |

A typical quantification of these advantages is given in table 1, which shows an analysis of UV active components in hydrocarbons streams. Converting to a robotic procedure improved the analysis time and turnaround time by about a factor of five, reduced the cost by a factor of almost four, and increased the precision by a factor of better than three.

**ROBOTIC NITROGEN DETERMINATION**

- Originally Installed in 1983
  Two Modifications Since That Time
- $120,000 Total Cost of Robot and Analyzers
  - 4 Month Payback Time
- Operation of 8–18 Hours/Day, Maintenance and Set-up of 1 Hour/Day, 2-Week Training Time for New Personnel

*Figure 6.*

**CARBON/HYDROGEN ROBOT PROCESS FLOW DIAGRAM**

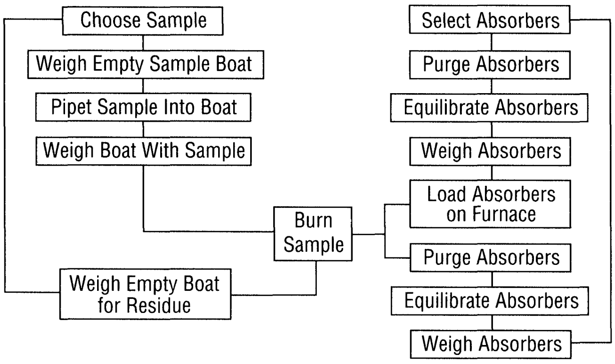

*Figure 7.*

One of the earliest robotic applications was a nitrogen determination in which the material was combusted and the resulting nitric oxide determined by chemiluminescence (figure 6). Although the initial cost of the system was around $120 000, the robot paid for itself within four months. It has since run around 40 000 analyses, has had two upgrades since its initial installation, and requires no more than about one hour of set-up and maintenance time per day.

Somewhat more complex is the robot designed and built for gravimetric carbon hydrogen determination. The process flow diagram is shown in figure 7. From the process flow diagram, however, it should be clear that complex and interrelated processes have been effectively reduced to practice in making this robot operational. It now routinely handles all of the authors' carbon and hydrogen analyses.

Most of the previous examples have involved robots to handle liquids, but robots will also successfully handle solids. Figure 8 is a photograph of a robot which prepares samples for X-ray diffraction. It grinds, sonicates, and centrifuges rock samples to produce uniform particles which are then subjected to XRD. This system was custom built by Zymark and came in close to target on both cost and delivery; it was functioning two weeks after it had been delivered.

A major application for robotics is in the environmental analysis area. One of the authors' early robots was designed to determine total suspended solids on manufacturing plant aqueous effluent (figure 9). This robot application was installed both in a research laboratory and in a refinery, and successfully handles this routine environmental analytical procedure.

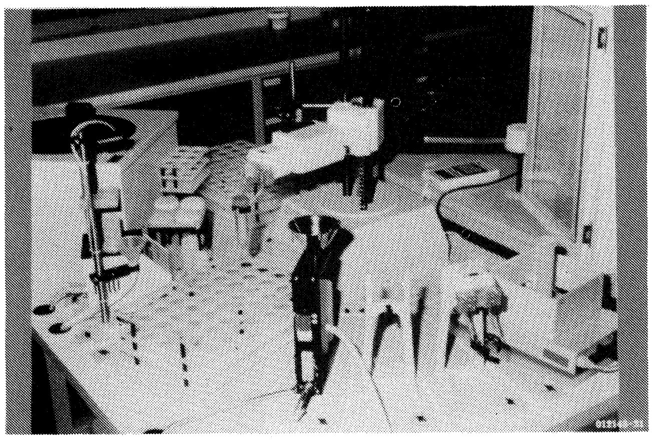

*Figure 8.*

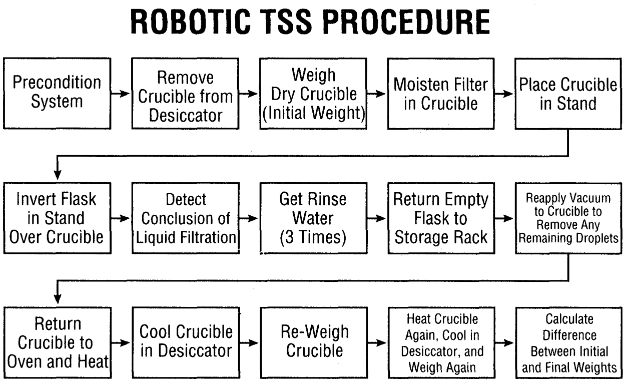

*Figure 9.*

While many robots are designed for a specific application, the authors built some which are multi-tasking. One of these handles six different kinds of titration methods, incorporates on-line quality control charting as an integral part of the system, and tracks the disposal of its own waste solvent (figure 10).

Quality control and quality assurance can easily be built into the robotic software. For example one of our robots will suspend the analysis if the quality control sample is outside acceptable limits.

Some of the more exciting opportunities arise from the interaction of people with robots. In the example shown in figure 11, there was an urgent unforeseen demand to find traces of residual metal in a chemical product. The number of necessary analyses was suddenly very high and there was no time to use a standard approach to robotize the procedure. However, a laboratory assistant recognized that an existing robot had some idle capacity, that it

**MULTI-TASKING POTENTIOMETRIC TITRATIONS**

- Developed by Third Party to Shell Specifications
- Several Different Titration Methods Included
- On-Line Quality Control Charting Is An Integral Part of the System
- Waste Management

*Figure 10.*

was particularly skilled at making dilutions, and that the analytical method could be modified to take advantage of these attributes. Within a few weeks, these modifications had been developed and tested and the problem was successfully solved.

In table 2 some of the major robotic applications installed in Shell during the last five years are displayed. Robots now run about 23 000 routine samples a year, or about one-fifth of the total load of quantitative samples. Over the next few years this proportion will increase significantly. These techniques are also being used successfully at some of Shell's refineries and chemical plants (table 3).

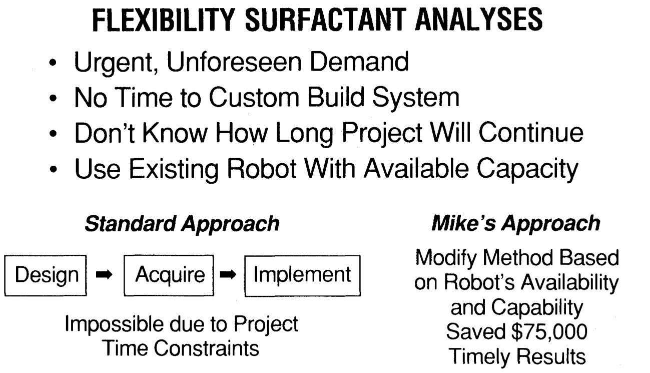

*Figure 11.*

In addressing barriers to robotic implementation, a common concern seems to be whether or not there is enough space to contain the robotic equipment (figure 12). While this is a legitimate concern, it can often be mitigated by innovative design, especially stacking of the robots in multi-level enclosures (figure 13).

*Table 2. Shell's robotic installations.*

| Method | Year | Number of analyses/year |
|---|---|---|
| Nitrogen | 1983 | 5700 |
| Ni/V; UV active | 1984 | 4500 |
| Total suspended solids | 1987 | 2000 |
| Detergency testing | 1987 | 5000 |
| Potentiometric titrations | 1987 | 1000 |
| Molecular weight | 1988 | 600 |
| X-ray diffraction | 1989 | 900 |
| Carbon/hydrogen | 1989 | 3000 |

*Table 3. Shell's robotic applications at refineries and chemical plants.*

| Method | Location | Year | Number of analyses/year |
|---|---|---|---|
| Biochemical oxygen demand and total suspended solids | Deer Park | 1987 | 3600 |
| Simulated distillation | Woodriver | 1987 | 1000 |
| Hydrogen by NMR | Woodriver | 1987 | 700 |
| Biochemical oxygen demand | Woodriver | 1989 | 3300 |

Another legitimate concern is how to provide in-house technological support for the robotic systems. At Shell there are focal points whose special expertise makes them helpful to a large number of prospective robot users.

Some imaginary barriers include the simple fear of the unknown, which can be mitigated by education and moral support. It is interesting to note that despite the installation of robotic systems, total employment and staffing of the analytical function at Shell has tended to go up rather than down over the period.

The role of management in implementing robotics is shown in figure 14. Basically, managers have to ensure that a process is in place for identifying those applications with economic incentive and high probability of success, and to provide resources and priorities to ensure that implementation proceeds.

**BARRIERS**

| | *FIX* |
|---|---|
| ***Real*** | |
| Space | Stacking, Remodel |
| Inhouse Support | Hire/Train Personnel |
| ***IMAGINED*** | |
| Fear | Education/Support |
| Job Security | Expand Job Opportunities |

*Figure 12.*

An equally important role is that of the technical staff who have to generate options of where robots can be used and to ensure that realistic assessments are made of success, cost, and timing (figure 15). They also need to ensure that their management does not have an erroneous bias, either positive or negative, of the problems or benefits of robotization.

A key role is also that of vendors (figure 16) who have to provide realism in cost and delivery times and work together with customers to extend robotics into new unit operations with broad applicability.

The authors' perception of the future is shown in figure 17. At present most of Shell's robots are self-correcting, in that they can be programmed to respond to particular kinds of errors. Newer robots will probably become successively more involved in making on-line decisions, and could ultimately evolve into expert systems.

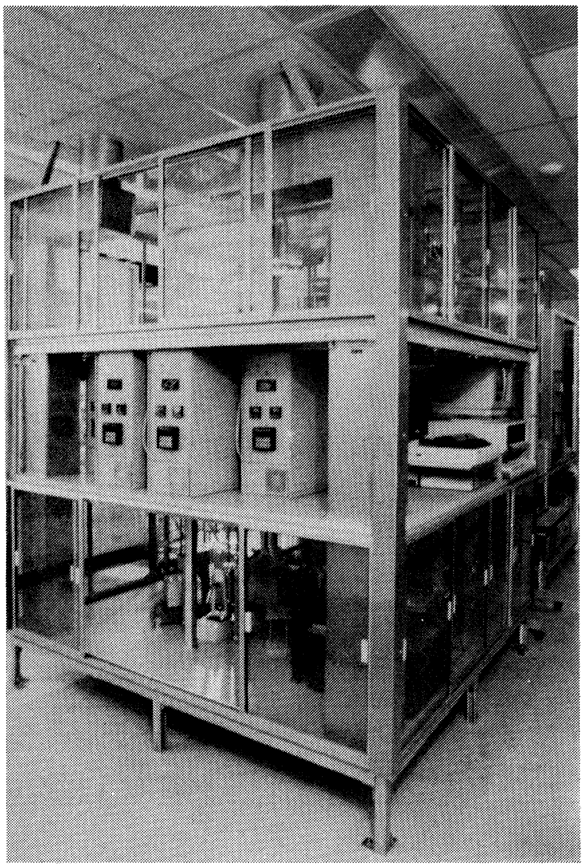

*Figure 13.*

**MANAGEMENT'S ROLE**

- Ensure Process in Place for Identifying Target Applications
  - Incentive
  - Probability of Success
  - Strategic Priorities
- Provide Necessary Resources
  - Programming
  - Capital
  - Moral Support
- Ensure That Project Has Appropriate Priority Within Department Goals and Strategy

*Figure 14.*

### TECHNICAL STAFF'S ROLE

Generate Plenty of Options

Provide Realistic Assessment of:
- Technical Success
- Cost
- Resources Required

Communication
- Ensure Management Understands Both Problems and Benefits

*Figure 15.*

It would be a natural development to integrate robots with the logistics of the authors' sample request system and to automatically record their output in central data archives.

Walk-up capability could be provided for customers by robots which could read procedures and sample designations from bar codes and perform and report the corresponding analyses.

### VENDOR'S ROLE

Provide Realistic Cost and Delivery Times for Both Components and Systems

Work Together With Customers To Extend Into New Unit Operations With Broad Applicability

*Figure 16.*

The number of quantitative analyses in which robots are involved will probably increase significantly over the next few years. It may well exceed 50% of the authors' work load by 1995.

The use of robotic facilities could improve reproducibility between different laboratories as typically measured by round-robin testing such that a tighter control of product specifications could be developed. Similarly, they will probably be increasingly used in regulatory standardized tests, such as those required to meet environmental regulations.

### FUTURE

- Self-Correcting Decision-Making Expert Systems
- Integration Into Sample Request System and Central Data Archives
- Customer Walk-up Capability
- Number of Quantitative Robotic Analyses:
  1990 20%
  1995 >50%
- Increase in Required Standardized Tests, e.g., Environmental Regulations
- Improved Reproducibility Between Laboratories
- As System Complexity and Customization Increase, There May Develop a Gap Between What Users Would Like to Have and What Vendors Would Like to Provide

*Figure 17.*

Finally, as robot users develop more familiarity with the capabilities of the systems, more and more complex applications will probably be requested. Vendors may prefer to select less complex, high volume applications which may have greater application volume potential. Between these two aspirations, there may develop a presently unfulfilled need for some third party to bridge the gap between what the users would like to have and what the vendors would like to provide.

In conclusion, experience at Shell with robotics has been very positive. In every case, benefits can be identified that were not foreseen in the original justification of the robotic equipment. This area is therefore recognized as one of high strategic priority, and will be developed with all deliberate speed.

